# “This is Slowly Becoming my Interest…”: The Understanding of Leisure and Preferences for Leisure Activities of People Receiving Adult Day Services

**DOI:** 10.1177/01640275231221162

**Published:** 2023-12-12

**Authors:** Mike Rommerskirch-Manietta, Christina Manietta, Daniel Purwins, Kimberly Van Haitsma, Katherine M. Abbott, Martina Roes

**Affiliations:** 1 172279 Deutsches Zentrum für Neurodegenerative Erkrankungen (DZNE), Witten, Germany; 2 School of Nursing Science, Faculty of Health, Witten/Herdecke University, Witten, Germany; 3 College of Nursing, 8082 Pennsylvania State University, University Park, PA, USA; 4 Department of Sociology and Gerontology, 6403 Miami University, Oxford, OH, USA; 5 Scripps Gerontology Center, 6403 Miami University, Oxford, OH, USA

**Keywords:** long-term care, adult day care, community, dementia, person-centered

## Abstract

Currently it is unknown what people receiving adult day services (ADS) understand as leisure and the activities they prefer remain unknown. To address these gaps, we investigated the understanding of leisure of people receiving ADS. We conducted semistructured interviews with 15 people receiving ADS in Germany. Interviews were analyzed using reflexive thematic analysis. *The sweet bitter symphony* emphasizes the sensations that shape participant’s understanding of leisure. *Young, wild & free*! describes the types of preferred activities. *Is this our last tango?* refers to the barriers. *Anchors aweigh! the [ongoing] voyage* describes the process by which leisure is transferred from private domain to the ADS environment. *The beginning is the end is the beginning* illustrates the paradox of understanding the ADS as offering a form of leisure and the adaptation to engage in nonpreference-based activities. Our findings indicate the importance in offering leisure activities that enable preference-based engagement in the ADS.

## Introduction

Community-based long-term care environments such as adult day services (ADS) represent an important care setting that offers activities to support the health and well-being of older people with various care needs, such as those resulting from the symptoms of dementia, as well as their families. The importance of such environments is due to the fact that ADS provide support for older people to age in place, which is their care preference, followed by assisted living and continuing care retirement communities (Kasper et al., 2019; Sadarangani et al., 2021a). For instance, ADS offers family caregivers the opportunity to remain employed, to experience respite and relief, and thus to stabilize the care situation at home. This benefit was particularly evident in the “real life setting” during the first year of the COVID-19 pandemic, when centers providing ADS were closed and, for example, family caregivers reported a decline in their relative´s well-being and needed to reduce their working hours to care for their relative (Gaugler et al., 2021).

In contrast to other long-term care environments such as nursing homes, ADS in the US is characterized by the fact that it is used by people who are younger (of an estimated 251.100 clients enrolled in ADS in 2018, 65% were aged 65 or older, 39% were below the age of 65, 42% were aged 65–85, and 19% were aged 85 or older), and more ethnically and racially diverse (22% Hispanic, 17% non-Hispanic black, and 16% non-Hispanic other) (Lendon & Singh, 2021). Despite the fact that the statutory care insurance in Germany covers a large portion of the costs of ADS, no representative detailed country-wide data have been provided for Germany (which featured an estimated 139.200 clients enrolled in ADS in 2019) (Destatis, 2020).

The lack of up-to-date data in Germany is due to the fact that little research has been conducted in the field of ADS. This research gap appears similar to the situation in the US (Li et al., 2022), and extant research in ADS has primarily focused on the establishment of standardized measurements for key outcomes for clients, caregivers and policymakers, such as client well-being, or the development/testing of study designs for investigating the effectiveness of ADS (interventions) for clients and their caregivers (Roth et al., 2020; Sadarangani et al., 2021b). In addition to these research activities, person-centered care in the context of ADS now appears to be receiving attention from researchers (Sadarangani et al., 2021a).

Person-centered care is associated with a high quality of care in the long-term care environments (National Academies of Sciences, 2022). One important factor for person-centered care is the need for services to be based on the preferences of their clients (Santana et al., 2018).

A focus on preference-based leisure activities thus seems to be one way of addressing well-being as a key client outcome (Sadarangani et al., 2021b) and to operationalize person-centered care (Van Haitsma et al., 2020) in the context of ADS.

### Achieving Well-Being Through Preference-Based Leisure Activities

For people to maintain or improve their individual well-being, it is important for their personal growth that they are able to satisfy their intrinsically motivated psychological needs (Deci & Ryan, 2000). Based on the self-determination theory (SDT) developed by Deci and Ryan (2000), these needs are (a) competence, e.g., mastering tasks; (b) relatedness, e.g., experiencing a sense of belonging and connection with other people; and (c) autonomy, e.g., having control over one’s own behaviors and goals. Deci and Ryan (2000) suggested that activities of interest (e.g., leisure activities) are one way in which people can address and satisfy their psychological needs, thus leading to the assumption that psychological needs underlie the individual’s understanding of leisure.

**Figure 1.**
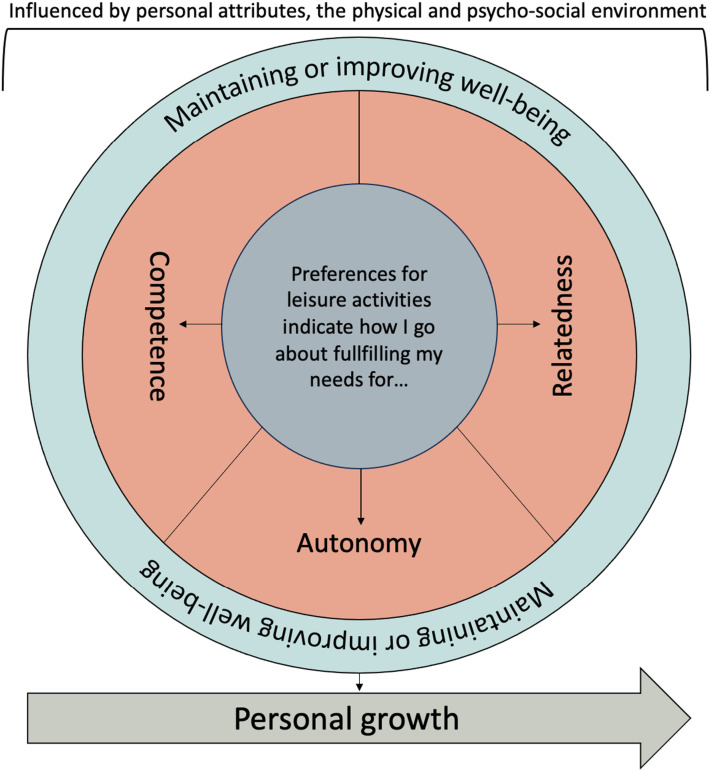
Modified self-determination theory and preference-based model of care in the context of leisure (Deci & Ryan, 2000; Van Haitsma et al., 2020).

Van Haitsma et al. (2020) incorporated these concepts into their preference-based model of care, emphasizing the fact that people’s individual preferences are indicators of how they want their psychological needs to be satisfied. Furthermore, it is noted that taking preferences into account in the context of service planning influences clients’ behavior in such a way as to promote active participation in activities. Especially for older people with a variety of care needs, who may have vulnerabilities (personal attributes) that can lead to barriers to preference-based care. For example, known barriers include the knowledge on the part of others (e.g., care providers) regarding individual preferences and necessary support from the environment (e.g., physical and psycho-social) with respect to engaging in their preferred activities (Van Haitsma et al., 2020) (Figure 1).

We propose that the care environment of ADS could serve as such a person-centered environment that can support people, who experience barriers with respect to participating actively in leisure activities that are important to them by offering preferred leisure activities to promote their clients’ leisure satisfaction, satisfy their psychological needs, and thus maintain or improve their well-being.

However, the ways in which people receiving ADS understand leisure and the leisure activities that they prefer remain unknown. To address these research gaps, we investigated the following overarching research question: “What do people receiving ADS understand as leisure?”

## Research Design

To answer our research question, we conducted semistructured interviews in centers providing ADS in Germany. These interviews were embedded in a process of concept mapping (Kane & Trochim, 2007) and were thus part of a larger participatory study (2020–2023) with the aim of developing an instrument for assessing preferences in the context of ADS (Rommerskirch-Manietta et al., 2021a, 2021b, 2022, 2023). Ethical approval was granted by the ethics committee of the Witten/Herdecke University. For the reporting of our interviews and to ensure rigor, we used the Consolidated Criteria for Reporting Qualitative research (COREQ): A 32-item checklist for interviews and focus groups (Supplementary Material Table 1).

### Recruitment and Participants

Due to the embeddedness of the interviews in the process of concept mapping, our aim was to recruit at least thirteen people receiving ADS. For concept mapping, a minimum of 10 people is recommended to obtain a good working framework and a diversity of opinions on a topic (Kane & Trochim, 2007). Since we assumed that not all participants would be present during each step of the concept mapping, e.g. due to hospitalization, to ensure that no less than 10 participants were involved in step, we increased the minimum number for recruitment to 13.

The focus of recruitment was on people receiving ADS who exhibited diverse characteristics (e.g., age, gender, cultural background, cognition). Accordingly, we chose to employ a purposive sampling technique (Patton, 2014). People receiving ADS were included if they: (a) used the ADS at least once per week or four times per month and (b) were able to verbally report on, for example, their understanding of leisure and preferences regarding leisure activities.

For the recruitment of potential ADS centers for participation, we informed our practice partners from a former study (Rommerskirch-Manietta et al., 2021a, 2021b, Stacke et al., 2020, 2021) of the upcoming project via email. Based on their feedback, we partnered with three different ADS centers who agreed to participate by helping us recruit their clients. After the three ADS centers agreed to participate, two researchers (MRM, MR) conducted virtual kickoff meetings via Zoom (Zoom Video Communications, 2022) with the nursing staff to inform them of and discuss the objectives of the study, our predetermined list of inclusion and exclusion criteria, and the focus and process of the recruitment of potential participants. Since the nursing staff were familiar with the people for whom they cared and since we had already gained experience with our practice partners in previous projects, we decided against a cognitive screening because this approach would have caused additional burdens for the participants and would have extended the data collection by another day per participant (First day: cognitive screening; second day: interview). Subsequently, participating ADS centers received recruitment flyers and posters from the research team via mail, and one researcher (MRM) conducted kickoff meetings in participating ADS centers to inform potential participants and to discuss the objectives of the project with them. A nurse working in the ADS centers, identified people receiving ADS who matched our inclusion criteria, and asked them to participate in the study. If they agreed to participate, the responsible nurse obtained written informed consent from them and, when necessary, their legal guardians. Because of this recruitment process, the refusal rate regarding participation in the interviews remained unknown to us. Detailed descriptions about our ethical prognosis and prevention, and ongoing consent is described in our study protocol (Rommerskirch-Manietta et al., 2021). Information regarding participants was then forwarded to one researcher (MRM) via e-mail, and appointments for the interviews were made based on the participants’ preferred days.

### Data Collection

We drafted the interview guide for our semistructured interviews based on the framework developed by Kallio et al. (2016). The interview guide was developed first by one researcher (MRM) based on a narrative literature review with a focus on theoretical and empirical conceptions of leisure among older people, and the guide was subsequently discussed and modified in separate group meetings with the coauthors (CM, DP, MR) and the group leader responsible for methods in health care research at the Deutsches Zentrum für Neurodegenerative Erkrankungen in Witten. Subsequently, a second draft of the interview guide was discussed with two nurses (a geriatric nurse and a nursing director) working at the participating ADS centers, and changes related to the wording of the questions were made. This draft was then pretested in a field test with a person receiving ADS who was recruited from a participating ADS. The purpose of this pretest was to investigate the interview’s points of intelligibility as well as the relevance of the questions to be asked (Kallio et al., 2016). As a result, no modifications were made to the interview guide, and the pretest was included in the analysis. The finalized interview guide is provided in Supplementary Material Table 2.

One researcher (MRM) conducted semistructured, face-to-face interviews in the participating ADS centers between February and July 2022. This researcher (white, cisgender male) has a professional background as a geriatric nurse and experience in conducting interviews with older people both with and without dementia. Participants were free to choose where in the ADS the interviews were conducted. In addition, if desired by the participants, related persons, such as family members or staff members with whom they were close, were allowed attend the interviews. Two participants chose to take advantage of this possibility. Interviews were recorded using an audio recorder. After the interviews were conducted, field notes were made, and additional demographic characteristics of the participants and contextual information regarding the ADS were collected by the same researcher.

### Data Analyses

Demographic characteristics and contextual information were analyzed descriptively, and participants’ names were pseudonymized. All interviews were professionally transcribed verbatim by a transcription agency, with, for example, inflections, breaks, and pauses being noted. One researcher (MRM) randomly checked the transcripts against the audio recordings for accuracy.

The transcribed interviews were then analyzed using a qualitative software program (MAXQDA 2022 Plus) (VERBI Software, 2022) via reflexive thematic analysis. This analysis was guided by the six phases described by Braun and Clarke (2020). We chose to conduct a reflexive thematic analysis to respect and reflect on the subjective understanding of leisure expressed by the most vulnerable participants and to take into account our own theoretical influences and presumptions (Deci & Ryan, 2000; Van Haitsma et al., 2020). We aimed to obtain an experiential understanding of the phenomenon of leisure, and so we focused on participants’ interpretations, views, and experiences of the phenomenon (Braun & Clarke, 2019). Based on the characteristics of the interview participants (e.g., a diagnosis of dementia), we decided to use a mainly inductive latent coding approach featuring preselected theory-driven deductive categories. The detailed analytical process we conducted is described in Table 1.

**Table 1.** Analytical Process.

Six phases of reflexive thematic analysis according to Braun and Clarke (2020)	Description
Phase 1: Data familiarisation and writing familiarisation notes	⁃ During the first phase, two researchers (MRM, CM) (both white, cisgender male and female) with experience in qualitative analysis separately read and reread all manuscripts, familiarized themselves with the data material, and wrote notes containing initial ideas.
	⁃ In a subsequent meeting, initial coding considerations, and ideas were discussed between these two researchers.
Phase 2: Systematic data coding	⁃ During the second phase, initial codes for the understanding of leisure and barriers for preferred leisure activities were derived deductively based on the psychological needs (competence, relatedness, and autonomy) emphasized by the SDT and the preference-based model of care (personal attributes and physical, psycho-social environment) (Deci & Ryan, 2000; Van Haitsma et al., 2020).
	⁃ Subsequently, one researcher (MRM) initiated the coding process and deductively/inductively coded any data item in the transcripts that seemed to be relevant to our research question.
	⁃ This researcher’s coding was discussed, reflected on, and revised as necessary with the help of another researcher (CM) during weekly meetings over a period of eight weeks.
	⁃ Since no new codes were identified based on the analysis of the final two interviews, we assumed that we had reached data saturation.
Phase 3: Generating initial themes from coded and collated data	⁃ Third, initial themes were jointly constructed (MRM, CM) based on the coding, and codes were merged accordingly.
	⁃ Following the method developed by Braun and Clarke (2019), we creatively constructed our themes. For this purpose, we chose metaphors inspired by song names that are catchy and capture the analytical essence of the themes.
	⁃ Finally, we summarized the different themes in a broader frame.
	⁃ In addition, an initial thematic map was created.
Phase 4: Developing and reviewing themes	⁃ During the fourth phase, one researcher (MRM) checked all possible themes and their related coding for incongruence and relevance with respect to our research question.
	⁃ Following this process of reviewing and adjusting the possible themes and the initial thematic map, the two researchers (MRM, CM) met once per week for another period of four weeks to discuss the results of this process.
Phases 5 and 6: Refining, defining, and naming themes, and writing the report	⁃ During the fifth and sixth phases of the reflexive thematic analysis, summary themes and the thematic map were presented to, discussed with, and finalized and named by the research team, resulting in the production of this written manuscript with selected quotations and a thematic map.
	⁃ Finally, the results were presented and discussed in a meeting with ADS stakeholders. An illustration of our coding process is provided in Supplementary Material Figure 1.

## Results

### Sample Characteristics

In total, we conducted 15 interviews with people receiving ADS. The length of the interviews ranged from 18 to 75 min, with a mean length of 41 min. Most participants reported being female (*n* = 11) and their ages ranged from 62 to 92 years. The majority of participants had a diagnosis of dementia (*n* = 11) or another form of cognitive and/or psychiatric disease (*n* = 3). The detailed characteristics of the participating ADS centers and people receiving ADS are described in Tables 2 and 3.

**Table 2.** Characteristics of the Participating ADS Centers.

Variables	Adult Day Service 1	Adult Day Service 2	Adult Day Service 3
Provider	Non-Profit	Non-Profit	Non-Profit
Location	Urban	Urban	Urban
Number of employees	⁃ 2 full-time	⁃ 1 full-time	⁃ 3 full-time
	⁃ 3 part-time	⁃ 5 part-time	⁃ 2 part-time
Profession of employees	⁃ Nurses	⁃ Nurses	⁃ Nurses
	⁃ Nursing and activity assistants	⁃ Activity assistants	⁃ Activity assistants
Number of clients	⁃ Daily: 15-19 clients	⁃ Daily: 14 clients	⁃ Daily: 14 clients
	⁃ Overall registered: 32 clients	⁃ Overall registered: 40 clients	⁃ Overall registered: 34
Business hours	Monday to Friday from 10:00 a.m. – 6:00 p.m.	Monday to Friday from 8:00 a.m. – 4:00 p.m.	Monday to Friday from 8:00 a.m. – 4:00 p.m.
Aim	⁃ Maintaining the client’s independence	⁃ Social inclusion	⁃ Spending the day in company
	⁃ Allowing clients to remain in their own homes	⁃ Preservation and promotion of the clients’ individual resources	⁃ Relieving the burdens of relatives
Services	⁃ Nursing and social care	⁃ Nursing and social care	⁃ Nursing and social care
	⁃ Care counseling	⁃ Care counseling	⁃ Care counseling
	⁃ Transportation	⁃ Transportation	⁃ Transportation
Daily schedule	⁃ 10:00 a.m.: Welcoming the clients and breakfast	⁃ 8:00 a.m.: COVID-19 test and breakfast	⁃ 8:00 a.m.: COVID-19 test and breakfast
	⁃ 11:00 a.m.: Activities such as newspaper roundup, crosswords, and physical exercises	⁃ 9:30 a.m.: Activities such newspaper roundup, group games, and biography work	⁃ 9:30 a.m.: Activities such newspaper roundup, biography work, and physical exercise
	⁃ 12:00 p.m.: Lunch	⁃ 11:45 a.m.: Motto of the day	⁃ 12:00 p.m.: Motto of the day and lunch
	⁃ 1:00 p.m.: Naptime or self-selected activities	⁃ 12:00 p.m.: Lunch	⁃ 1:00 p.m.: Naptime or activities such as board games, walking, and painting
	⁃ 3:00 p.m.: Coffee and cake	⁃ 1:00 p.m.: Naptime or activities such as painting, round-table discussions, and rickshaw rides	⁃ 3:00 p.m.: Coffee, cake, and group activities
	⁃ 4:00 p.m.: Activities such as bowling, bingo, and puzzles	⁃ 3:00 p.m.: Coffee, cake, and group activities	⁃ 3:45 p.m.: Goodbye and drive home
	⁃ 5:15 p.m.: Dinner	⁃ 3:45 p.m.: Goodbye and drive home	
	⁃ 5:45 p.m. – 6:00 p.m.: Goodbye and drive home

**Table 3.** Characteristics of the People Receiving ADS, Who Participated in This Study.

Name^a^	Age	Gender	Origin	Education	Former Work	Living With Other People in the Same Household	Degree of Care Needed^b^	Dementia Diagnosis (or Other Cognitive Diseases)	Tenure Receiving ADS	Days Per Week Receiving ADS	Self-Reported Reason for Receiving ADS	Additional Care Services
Ilse	74	Female	Germany	9^th^ grade secondary school	Industrial clerk	Husband	5	Yes	2 years	4	I don’t want to move into a nursing home	⁃ Home care
												⁃ Social services
												⁃ Occupational and physical therapy
Ludwig	89	Male	Germany	8^th^ grade secondary school	Car mechanic	Wife	3	Yes	4 years	3	I need to be around people; otherwise, I don’t feel well	None
Adelia	75	Female	Portugal	4^th^ grade primary school	Post office worker	No	4	No	4 months	2	My daughter doesn’t want me to be home all day alone	⁃ Home care
												⁃ Speech and physical therapy
Wolfgang	64	Male	Germany	College; master’s degree	Architect	No	4	Yes	5 years	4	I want to fill my days with activities and entertainment	None
Heinrich	85	Male	Upper Silesia	7^th^ grade secondary school	Welder	⁃ Wife	4	Yes	1.5 years	4	I would like to participate more in activities	None
						⁃ Daughter						
						⁃ Son-in-law						
						⁃ Grandchildren						
Stenzel	79	Male	Silesia	Basic primary and secondary school	Industrial mechanic	No	3	Yes	1 month	3	I feel lonely at home	None
Annette	65	Female	Germany	Basic primary and secondary school	Saleswoman	⁃ Daughter	4	(Brain aneurysm)	9 months	1	My children are working, and I am alone during the day. I have already forgotten to turn off the stove once.	None
						⁃ Son-in-law						
						⁃ Grandchildren						
Rita	92	Female	Germany	Basic primary and secondary school	Nurse	No	2	Yes	11 months	3	I want to be among people and not alone	None
Jutta	69	Female	Germany	Basic primary and secondary school	Housekeeper	No	3	(Intellectual disability)	6 months	3	I need entertainment, and I’m alone at home	None
Regina	84	Female	Germany	Basic primary and secondary school	Hairstylist	No	2	Yes	2 months	2	Getting well again, I got bored at home, and my daughter signed me up after my stroke	⁃ Home care
												⁃ Occupational therapy
Elsbeth	81	Female	Germany	No information	School office	24-h nursing assistant	3	Yes	3 months	2	A change from my everyday life	In-home 24-h nursing assistant
Käthe	85	Female	Germany	2-year business school	Secretary	Husband	2	Yes	4 months	3	I always wake up at night and feel restless. My husband needs time to relieve himself	Home care
Klaus	79	Male	Germany	Basic primary and secondary school	Locksmith	Wife	3	Yes	2 years	4	I was bored at home, and then my wife brought me here	None
Edith	80	Female	Germany	High school	Secretary	Husband	3	(Depression, Mania)	2 years	3	So that my husband can work in peace	None
Sieglinde	85	Female	Germany	Basic primary and secondary school	Housekeeping	Daughter	3	Yes	5.5 years	3	I need variety; I’m bored at home.	None

^a^Pseudonymized.

^b^Ranges from zero to 5; higher scores indicate higher dependency on care.

### Overview

**Figure 2.**
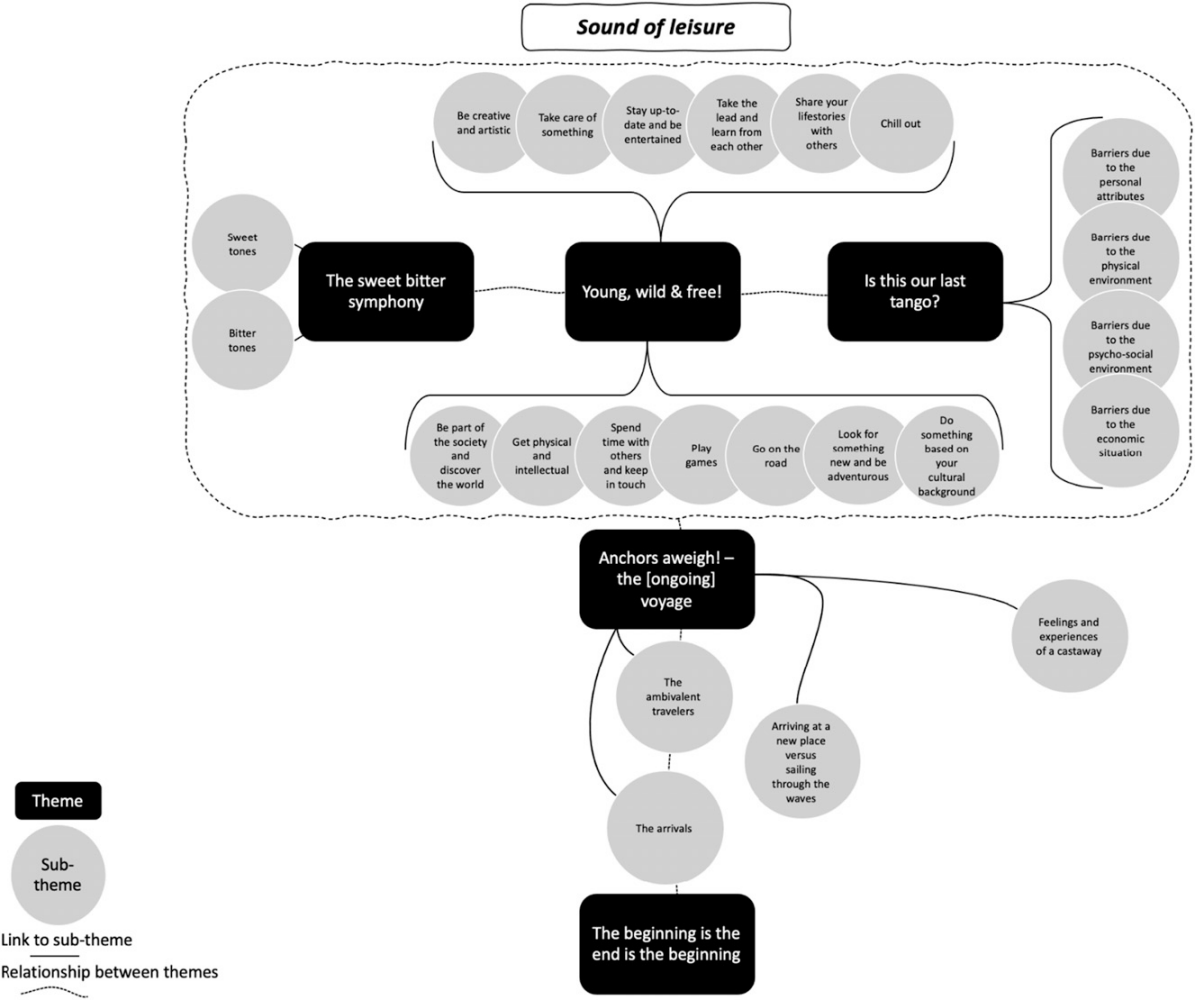
Thematic map.

Overall, we were able to construct five different themes based on our data, which were tied together in a broader frame as the “sound of leisure” in the lives of the participants (Figure 2).

Participants highlighted the different senses and feelings they had associated with leisure throughout their lives, which had thus shaped their understanding of leisure. We present this in the context of our first theme, *the sweet bitter symphony*.

These different senses and feelings are mainly related to the individual preferred leisure activities in which the participants engage in or have engaged throughout their lives. We present this broad range of preferred leisure activities in the context of our second theme, *young, wild & free!*

In addition to their preferences for various leisure activities, participants also described the barriers they face with regard to those preferred leisure activities. We present these barriers in the context of the third theme, *is this our last tango?*

Our fourth theme, *anchors aweigh! – the [ongoing] voyage*, summarizes participants’ descriptions of their understanding of the extent to which the ADS is viewed as a place for leisure.

Finally, participants in the interviews highlighted a range of leisure activities provided by the ADS. The paradox between the leisure activities provided by the ADS and the preferences of the participants in light of their understanding of the ADS as a place for leisure is presented in the context of our final constructed theme, *the beginning is the end is the beginning*.

### Theme 1: *The Sweet Bitter Symphony*

The sweet bitter symphony was chosen as a metaphor because participants used their voices (symphony) to describe their understanding of leisure in both positive (sweet tones: competence, relatedness, autonomy) and negative (bitter tones: incompetence, unrelatedness, heteronomy) senses as well as their corresponding feelings.

#### Sweet Tones

In terms of sweet tones, participants understand leisure as pertaining to their ability to gain or affirm their personal competencies. This includes participating in *competitions* such as sport tournaments and experiencing *success*, *mastering* their leisure activities, for example, certain techniques for bowling, *learning* new things such as how to use a new machine to engage in craft activities, and being able to take *responsibility* for something or someone, for example grandchildren. In the context of mastering an activity, one participant described the following:

So, and most importantly, the dog listened to me, she [the shepherd dog] was trained; I could also let her run free, although, she had a pronounced hunting instinct. The terrier is like that; when he has a scent, he wants the wild animal. The shepherd dog, she lets herself be called back. She sees it and runs after it. And then I say: “Stop” or “Here”, no, and she came back, but the terrier told me here, I’ll run anyway. Yes, the terrier is a challenge. I would get a terrier again and again. (Regina)

In addition to the sense of competence, the participants also described that they understand leisure in terms of a sense of relatedness. In this context, the participants noted that they understand leisure in terms of *sharing the same interests* with other people and engaging in an activity together, for example, by going for a walk or a cycling tour with another person. Moreover, participants reported that for them, *feeling cared* for and *taking care* of others are also part of their understanding of leisure. One participant made the following comment with regard to taking care of others:

I have always stood behind my husband at the exit or next to my husband; we have said goodbye to the individual churchgoers with a handshake, and then I have always said, because they tell me from the congregation, no pastor's wife has ever done that. I say, that's not what matters; I'd like to do it. I know that there are many people who don't have a single form of communication during the day, and I thought that they should have exchanged a few words with each other. (Käthe)

Furthermore, participants understand leisure in terms of being *loved*, being *needed*, being *proud*, and being consistent with and following their own *self-concept*, such as their role as a grandmother. These aspects were particularly emphasized in the context of helping other people while engaging in one’s own leisure activities, such as crafting (“Sometimes people come and ask can you repair my apartment key?”) (Stenzel) or in the context of spending leisure with one’s family. One participant reported the following with respect to being loved:

Ben is the little one. “Grandma, I know, I know”. He’s always so smart; sometimes, he says “Grandma, are you okay?” And I say, “Yes, my darling, I’m very well”. “That’s nice”. That's always so nice, when he notices that I’m better; he climbs on my lap and hugs me and kisses me (laughs) that’s the nicest thing. (Annette)

Having *control* over what one wants to do was described by participants as an essential aspect of their understanding of leisure with respect to a sense of autonomy. For the participants, having control indicates that leisure is characterized for them by the free choice of activities, that they can be who they want to be, and that they can spend their leisure with the people with whom they want to associate. One participant described this as follows: “Freedom. So, what I want to do now, go out, make handcrafts, or talk with others” (Stenzel). Additionally, to this positive understanding of leisure, which is shaped by senses of competence, relatedness, and autonomy, participants also discussed a broad range of positive feelings they associate with leisure. These feelings include *entertainment*, *happiness*, *diversion*, *satisfaction, and fun*. As one participant reported, “We always have fun seeing the young animals; that’s very nice” (Edith). In addition, other positive feelings, such as *gratitude*, *calmness*, *vitality*, *recharge*, and feeling *sheltered* were mentioned.

I immediately think of a change of place. So yeah, I live here in the big city, and then I just have the urge to go to a smaller place, to feel more sheltered, to be on a bike, and to be in the water a lot. (Käthe)

#### Bitter Tones

In addition to these sweet tones, bitter tones were also identified in participants' understanding of leisure. These bitter tones include senses of incompetence, unrelatedness, and heteronomy. Accordingly, negative feelings are also associated with leisure. Participants noted that they understand leisure in terms of a sense of incompetence because they face certain *limitations* or have completely *lost* the ability to engage in their preferred leisure activities, for example, due to illness. As one participant noted: “Yes, that’s not possible for health reasons; if you have two ruined legs and you must stand, you can’t stand like that. Yes, my favorite dance was the tango” (Ludwig). Participants also mentioned that their leisure activities are sometimes *too challenging* and that they *failed* in competitions. Furthermore, they reported that during their leisure, their own competences are not recognized and that they to some degree feel *insignificant*.

Furthermore, participants noted that they understand leisure in terms of a sense of unrelatedness. One participant reported that *exclusion* from shared leisure activities with their spouse has occurred due to their own physical limitations.

I can walk for half an hour, then I can’t go on […]. Yes, it’s sometimes a bit sad, that’s true. But I fully understand that my husband must go out on his own. And he also needs that; he always needs the encounter with nature and with God and yes, and people, yes. (Käthe)

Moreover, participants reported that they understand leisure in terms of a sense of heteronomy because they feel *controlled* during their leisure and because *role expectations* restrict their free choose of activities.

But as a grandma you can’t say “no”, as a mother you can say “no”; it’s hard, but you can, but not as a grandma. As a grandma, you can’t. That’s not possible. Yes, I break their hearts when I say no. I tried it once, I said “No, now let me be alone for a while. I like to be alone for a little bit”; “But grandma, we just want to play”; I say: “Yes”. They couldn’t speak properly yet, but the little one still said “Grandma, p[l]ay” (laughs). I say: “Yes, we’ll play in a minute”. (Annette)

Negative feelings associated with leisure were noted by the participants, especially in relation to *sadness,* resulting from the realization that they are no longer able to perform certain activities.

Because of the pressure that is created in the head. As they told me at the hospital at that time, “You can do that, whatever, if you want to do that [playing the trumpet], do that, but you won’t enjoy it for long. We would like to forbid you to do that. But we are not allowed to do that. But for your own sake, please don’t! You won’t survive a second brain hemorrhage!” And then that’s statement enough. I was quite sad about it. (Wolfgang)

Finally, *exhaustion* and *physical pain* were also reported as a feeling associated with leisure: “I had sore muscles for at least a week after it [playing soccer], pooh” (Annette).

### Theme 2: *Young, Wild & Free!*

The metaphor of young, wild & free! was chosen to summarize the preferred leisure activities reported by the participants. As indicated by these song lyrics (Mars et al., 2011), participants’ preferences for leisure activities remained unaffected by barriers that influenced the performance of these activities in line with the motto “We don’t care […] living young, wild & free”; these preferences were also a basis for free self-expression. Participants reported preferences ranging from artistic and caring activities to social, inclusive, physical, gaming-based, and cultural activities. In addition, participants discussed the fact that the leisure activities they mostly preferred have not changed over their lifetime: “[In response to a question regarding changes in preferences,] no, no, no, no […] always the same!” (Edith) Participants noted that these preferences often arose from or are related to biographical events.

Where the war was, the front came closer and closer […] We drove and drove, and we didn’t know how far, how long. Until the soldiers told us that we had to drive away so that the Russians wouldn’t catch us […] then we came as far as Bavaria, to Niederaschau. Yes, we lived there in a gatehouse and that’s where I learned to play the harmonica. (Heinrich)

The broad range of different preferences noted by participants and further exemplary quotations are presented in Table 4.

**Table 4.** Preferred Leisure Activities.

Young, wild & free!			
***Spend time with others and keep in touch***	***Exemplary quotations***	***Be creative and artistic***	***Exemplary quotations***
⁃ Caring for others	⁃ Reminiscing: That was a very nice time, which we remember very fondly. (Ilse)	⁃ Distilling/brewing	⁃ Cooking: In the same way, I like to cook, and not just something, but properly, to satisfy my guests. (Wolfgang)
⁃ Being around other people	⁃ Be around other people: I like to be with people, and I also like to get in touch with them (Käthe)	⁃ Cooking	⁃ Handcrafts: Yes, handcrafting. So somehow making something, repairing something, things like that, yes. (Stenzel)
⁃ Talking with others		⁃ Baking	
⁃ Getting to know other people		⁃ Singing	
⁃ Building friendships		⁃ Playing instruments	
⁃ Writing to pen pals		⁃ Painting & drawing	
⁃ Playing pranks		⁃ Handcrafts	
⁃ Clubs		⁃ Mechanics	
⁃ Family and friends			
⁃ My children or other children			
⁃ Animals			
⁃ Reminiscing			
***Get physical and intellectual***	***Exemplary quotations***	***Be part of society and discover the world***	***Exemplary quotations***
⁃ Ball games	⁃ Bowling: Well, there were days when we bowled. That is still the case today. (Klaus)	⁃ Shopping	⁃ Attending festivals: We went to Halle in Thuringia every year for the Georg Friedrich Handel Festival. But that was also cancelled because of Corona. (Edith)
⁃ Tossing	⁃ Cycling: Yes, we are cyclists, yes. With my husband, yes. But now it’s been over for more than a year. And so we have completely normal wheels, Koga Miyata, which is a Dutch-Japanese company, and without a motor. (Käthe)	⁃ Going to restaurants	⁃ Tourism: Yes, we were, yes, were now in Austria and Switzerland. (Ludwig)
⁃ Bowling		⁃ Going to parks	
⁃ Mentally stimulating games		⁃ Sightseeing	
⁃ Swimming		⁃ Going to church	
⁃ Sailing		⁃ Attending sporting events	
⁃ Roller skating		⁃ Visiting fun fairs	
⁃ Dancing		⁃ Going to cabarets	
⁃ Gymnastics		⁃ Attending concerts	
⁃ Walking		⁃ Going to the opera	
⁃ Running		⁃ Attending festivals	
⁃ Cycling		⁃ Tourism	
⁃ Climbing			
⁃ Hand-picking			
⁃ Dog walking			
⁃ Dog sports			
⁃ Gardening			
⁃ Board games			
⁃ Quizzes			
⁃ Riddles			
⁃ Crossword puzzles			
⁃ Memory games			
⁃ Puzzles			
⁃ Cards			
⁃ Rolling dice			
⁃ Lotteries			
***Stay ** **up-** **to-** **date** ** and be entertained***	***Exemplary quotations***	***Take the lead and learn from each other***	***Exemplary quotations***
⁃ Reading	⁃ Reading: I like reading nonfiction most of all. With great pleasure. (Adelia)	⁃ Volunteering	⁃ Mentoring: How to dance the tango. She was so enthusiastic afterwards, in the middle of it, no, she really came because she understood everything. And she was so happy that I taught her properly. (Ludwig)
⁃ Listening to music	⁃ Watching TV: When they play, yes, for example Germany against Italy, which I watch anyway. Or Germany against England was yesterday or the day before. Yes. Yes, then I like that already. (Heinrich)	⁃ Mentoring	⁃ Learning new things: I can always learn something. And they told me a few tricks and you always learn something new. (Stenzel)
⁃ Watching TV		⁃ Initiating interest groups	
		⁃ Improving knowledge and skills	
		⁃ Learning new things	
***Chill out***	***Exemplary quotations***	***Look for something new and be adventurous***	***Exemplary quotations***
⁃ Relaxing	⁃ Relaxing: That I can then lie down for a while. (Käthe)	⁃ Trying out new things	⁃ Trying out new things: Yes, also to try out something new in terms of content. I’ve taken one or two pottery courses now recently. (Wolfgang)
⁃ Doing nothing	⁃ Doing nothing: Yes, you sit around like that sometimes. That’s good, too. (Rita)	⁃ Trying out everything	⁃ Trying out everything: Yes, if what is now proposed, and they say then what I do everything with. Whether I can then, I must then determine. But I’m trying everything. (Ludwig)
⁃ Taking time for myself			
⁃ Wellness			
***Go on the road***	***Exemplary quotations***	***Do something based on your cultural background***	***Exemplary quotations***
⁃ Driving car	⁃ Driving car: I like to drive. Sometimes I drive away, and sometimes I drive like a wild sow. (Stenzel)	⁃ Festivals	⁃ Sports: I’m looking forward to the World Cup when Portugal plays. (Adelia)
⁃ Using public transportation	⁃ Using public transportation: By bus, that’s leisure. The Rheinbahn also still goes everywhere. (Jutta)	⁃ Music	⁃ Consulting: And when something Bavarian comes up, they always say, “Then we’ll have to ask Edith” Because I come from Munich. (Edith)
		⁃ Sports	
		⁃ Consulting	
		⁃ Environment	
		⁃ Back home	

### Theme 3: *Is This Our Last Tango?*

In addition to their reported preferences, participants also discussed the barriers they face for their preferred leisure activities. We chose the following metaphor: is this our last tango? This choice was due to the fact that it is unclear whether participants’ preferred leisure activities can be engaged by them in due to the barriers that currently exist. The barriers noted by the participants include personal attributes such as *powerlessness* and *self-ageism*: “You know, I might be already too old [to play soccer]” (Heinrich). Additionally, barriers concerning the physical environment were also reported. Aspects such as *structures* and *COVID-19 restrictions* and their consequences were emphasized.

There are or were church services here; now, there is such a big break. Always rotating between Catholic and Protestant, one per week, and corona killed it all. For a long time, it just wasn’t allowed to happen. And I’m sorry to say, I feel like it’s fallen asleep, or it’s not being revived. (Wolfgang)

Furthermore, barriers resulting from the psycho-social environment, for example, different *interests* and forms of *stigma,* were mentioned by the participants. The following was described: “Yes, but then my wife would say, you’re sick [diagnosed dementia] and you want to do everything [playing soccer]” (Heinrich). Finally, participants’ economic situations were mentioned as a barrier, with a focus on their *financial capabilities* to engage in their preferred leisure activities. A detailed overview of all reported barriers and further exemplary quotations are provided in Table 5.

**Table 5.** Barriers for the Preferred Leisure Activities.

Is this our last tango?	
***Barriers due to the personal attributes***	**Exemplary ** ***quotations***
⁃ Physical	⁃ Powerlessness: I can’t stand that at all, yes. You’re exhausted because the disease is also so far advanced […] (Käthe)
⁃ Cognitive	⁃ Self-confidence: And learning a new instrument, I don’t dare to do that anymore. (Wolfgang)
⁃ Psychological	
⁃ Daily condition	
⁃ Self-ageism	
⁃ Powerlessness	
⁃ Lack of creativity	
⁃ Sense of purpose	
⁃ Self-confidence	
⁃ Loneliness	
⁃ Responsibility	
⁃ Pain	
⁃ Insomnia	
***Barriers due to the physical environment***	**Exemplary ** ***quotations***
⁃ Distance	⁃ Distance: I would have to drive from us to the stadium and that’s too far. That’s from one end to the other end. (Ludwig)
⁃ Inaccessibility	⁃ Relocation: I lived in the Eifel [a rural location] and I did my garden. My flowers. They were so beautiful. People all stopped and looked. [This participant now lives in a city] (Adelia)
⁃ Business hours	
⁃ Opportunities	
⁃ COVID-19 restrictions	
⁃ Relocation	
⁃ Structures and rules	
***Barriers due to the ** **psycho-** **social** ** environment***	**Exemplary ** ***quotations***
⁃ Interests	⁃ Stigma: It’s way too dangerous, so my kids don’t let me go out by myself. If they do, they go with me, and I don’t go [dancing] with my children. (Annette)
⁃ Retirement	⁃ Death: But for three years, I have no longer had a dog, which I unfortunately had to put down (Regina)
⁃ Denial	
⁃ Stigma	
⁃ Anonymity	
⁃ Interactions	
⁃ Capacity	
⁃ Aging	
⁃ Death	
***Barriers due to the economic situation***	**Exemplary ** ***quotations***
⁃ Financial capabilities	⁃ Financial capabilities: But I can’t afford it anymore [to work on cars] (Stenzel)

### Theme 4: *Anchors Aweigh! The [Ongoing] Voyage*

The metaphor of anchors aweigh! the [ongoing] voyage illustrates the process by which the location of leisure shifts from the private domain, which had previously been paramount, to the environment of ADS. This transition process (*voyage*) appears, on the one hand, mainly to have been completed for the participants (*the arrivals*), but on the other hand, it also appears to be ambivalent and still ongoing for some participants (*the ambivalent travelers*). Accordingly, as one participant reported, “[In response to the question of whether the ADS represents a place for leisure,] Yes, yes, absolutely […]” (Jutta). In contrast, the expressions of ambivalent travelers did not seem to provide a clear answer, or the time spent receiving ADS was associated with work, or the answer was more closely related to a process of negotiation and balancing.

So, there, I must confess, until now, not yet [i.e., understanding the ADS as a place for leisure] […] No, I have it dazzling, I have it good. I get food served, I have my bed, I can come and go as I please when I’m told, that’s clear; that’s already leisure, yes. But I always think that I must reach out to the community [in the ADS environment]. (Käthe)

These expressions can be understood to emphasize two distinct poles in relation to which the participants tend to situate themselves in their statements. The degree of attribution can be linked to the satisfaction or lack of satisfaction (*arriving at a new place versus sailing through the waves*) of participants’ psychological needs for competence, relatedness, and autonomy because of preference-based or nonpreference-based behavior. For example, regarding the satisfaction of participants psychological need for autonomy, the following was reported by an *arrival*: “I can practically make my own thing here” (Klaus). In contrast, the following was reported by one participant (an *ambivalent traveler*) regarding the lack of satisfaction of the psychological need for relatedness:

I like to be with people, and I also like to meet them. And the very first day, there was a screaming fight in the dining room; they were poisoning each other, I couldn't believe it. And then, I went home, and then I said to my husband that I don't know if I can get through this. No, but he tried so hard to get a place here at all, because it [the ADS] is in high demand. (Käthe)

Finally, participants noted that these unmet needs lead to negative feelings and experiences (*the feelings and experiences of a castaway*). These feelings and experiences include *waiting to go home* and a *self-image as a prisoner* (“So, if I don’t find out at all who among the fellow prisoners here is interested in what and who is not […]”) (Wolfgang), *weight loss*, *boredom*, and *apathy* (“[…] there’s nothing going on here; we sit here and stare at each other, and yeah, and so”) (Käthe); *loneliness* and *guilt* (“[…] I’d like to help or something, get cigarettes for [anonymized] because I know that. So, I wouldn’t do it again. The nurse reprimanded me […]”) (Bärbel) and *stress*, *and exhaustion* (“[…] I come back every evening completely exhausted […]”) (Käthe).

### Theme 5: *The Beginning is the End is the Beginning*

For our last theme we chose the metaphor of the beginning is the end is the beginning. This metaphor illustrates the paradox that the ADS is mainly understood as a place for leisure by the participants despite the fact that the leisure activities thus offered largely do not correspond to their understanding of leisure or reflect their preferences: “Yes, but you can’t find a teammate for that [the participant’s preferred activity of playing cards]” (Klaus) and “The games there are weird” (Ilse). Additionally, participants reported that they do not consider the ADS to enable them to resume their engagement in their preferred leisure activities: “No. I haven’t really thought about it yet either” (Wolfgang) or “Everything is wonderful. I do not know what else I can wish for” (Adelia). Instead, participants described a process of adaptation to the activities that are offered. This process, for example, cause participants to report that after some time, they no longer minded participating in these nonpreferred activities. “This is slowly becoming my interests [playing board games] […] then I do not mind anymore […]” (Wolfgang).

## Discussion & Implications

Our study explored the understanding of leisure among people receiving ADS. The understanding of leisure exhibited by people receiving ADS is shaped by their senses of competence, relatedness, and autonomy, as well as the opposites of these senses (incompetence, unrelatedness, and heteronomy). In addition, participants reported on positive and negative feelings they associate with their leisure. This study provides evidence indicating that the psychological needs identified by SDT can be understood as underlying the understanding of leisure exhibited by people receiving ADS. Furthermore, these psychological needs can be further specified in terms of other aspects. For example, we found that for people receiving ADS, competence, as a psychological need that is satisfied during leisure, is related to the aspects of competitions, success, mastering learning new things, and responsibility. This finding enhances the application of SDT in the context of leisure of people receiving ADS by providing concrete specifications of their psychological needs. Furthermore, our results highlight the importance of meaningful leisure for the ability of this group of people to maintain or improve their individual well-being and personal growth in accordance with SDT. In addition, our study provides further insights into the positive and negative aspects that must be addressed in the context of leisure activities aimed at preserving, for example, the cognitive and physical functioning of the clients of ADS as well as their mental health (Sala et al., 2019). Furthermore, self-reported feelings related to leisure, such as satisfaction, appear to be key outcomes for evaluating the leisure activities offered in the context of ADS and could enrich the current debate regarding outcome measurement in this context (Sadarangani et al., 2021b).

In terms of preferred leisure activities, the type of leisure activity in question does not seem to play the most important role in shaping preferences for one activity or another; rather, the meaning associated with the leisure activity in question appears to serve this function. This finding appears to be consistent with the conclusions of other studies focusing on older people beyond the context of long-term care (Myllykangas et al., 2002). An explanation for this finding might lie in the fact that the participants reported that their primary preferred leisure activities are strongly associated with and shaped by biographical events and do not change throughout the course of their lives. This characterization highlights the importance for ADS providers of knowing the person and their preferences regarding leisure activity. Instruments (e.g., Preferences for Everyday Living Inventory – PELI) with creative practical forms of implementation, such as the PAL cards (Abbott, Heppner, et al., 2021), are feasible ways of gaining knowledge about such preferences. For example, knowledge that someone prefers to engage in leisure activities with their grandchildren can be used to initiate conversations about the grandchildren, make gifts for the grandchildren, or invite the grandchildren to participate in joint celebrations in the ADS.

In terms of reported barriers, it is clear that simply being aware of these preferences is just the first step; barriers must be addressed in a participatory way by finding creative solutions to the challenges associated with preserving and promoting preference-based behavior. Achieving this goal requires the person’s own “voice” and willingness on the part of the care provider, which could be fostered by means of, for example, financial incentives, education and awareness, the implementation of feasible instruments into the workflow, and stakeholder engagement (Abbott et al., 2021a).

Enabling preference-based behavior also appears to be related to the extent to which the ADS is understood as a place for leisure as well as to the question of whether clients’ psychological needs are met. For example, although most participants reported that they understood the ADS as a place for leisure, they did not associate this environment with a realization of their preferences. Rather, they adapted themselves to what was offered, and the environment, in turn, influenced their preference-based behavior, thereby having a negative impact on their well-being. On the one hand, this finding empirically supports the preference-based model of care (Van Haitsma et al., 2020) in the ADS setting and, on the other, should be further investigated with the focus on the adaptation process described by the participants to the activities offered in the ADS. In this context, the Dual Process Theory of Assimilation and Accommodation seems to provide promising explanations as a theoretical foundation for further investigation of the underlying mind-sets of people receiving ADS regarding this phenomenon (Rothermund & Brandtstädter, 2019). Furthermore, it remains unclear whether this process of adapted nonpreference-based behavior can be reversed by offering and implementing leisure activities within ADS that are tailored to the preferences of the people receiving ADS.

Finally, our results, which focus on ADS in Germany, are the first to identify important aspects pertaining to the importance of leisure and preference-based leisure activity services in the German ADS environment. These results also seem to be confirmed in the international context. In the U.S., preference-based leisure activity services in the ADS context do not currently seem to be implemented, and activities are based on the needs and competencies of the people receiving ADS rather than their preferences (NADSA, 2023). Consequently, our study and findings appear to offer the opportunity to raise awareness of the field of ADS and this topic among international researchers with the aim of inspiring further international studies. This approach would address the lack of research activity focusing on the ADS environment (Li et al., 2022; Orellana et al., 2020) and further facilitate a cultural change in the ADS environment towards person-centered care on the international level (Sadarangani et al., 2021a). Furthermore, this approach would improve the care provided in terms of quality and the satisfaction of persons receiving ADS worldwide (Bucy et al., 2023).

### Limitations

Our study faces certain limitations. It should be noted that a selection bias may have impacted the study and influenced our results. On the one hand, we were only able to recruit ADS centers that were located in an urban area and on the other hand, our participants were recruited by nurses. This approach may have entailed that persons who habitually part in research were particularly likely to participate in this study. Consequently, other life experiences may have remained unreported. Despite the purposive sampling strategy that we employed, our sample does not appear to be very heterogeneous in terms of participants' cultural backgrounds and dementia diagnosis. However, due to a lack of statistical data and studies, it is still unclear how heterogeneous the population of people receiving ADS in Germany is and whether our sample reflects this population. Studies from Germany have reported that people with a migration background (e.g., people from Turkey) often do not use professional care services (Tezcan-Güntekin et al., 2022), and studies from the U.S. have shown that one of the four most frequently reported diagnoses among people receiving ADS is dementia (Lendon & Singh, 2021), which is reflected in our study sample.

### Conclusion

Offering leisure activities in the context of ADS that enable preference-based behavior appears to be a complex and challenging task in light of the understanding of leisure exhibited by people receiving ADS. Since the ADS is mainly understood by people receiving ADS as a place for leisure, it seems to be essential to create structures in the future to facilitate such an environment. This task includes investigating the needs of ADS providers with respect to addressing the various associated barriers and designing their services to promote preference-based care. Furthermore, future research should focus on the understanding of leisure exhibited by people from diverse cultural backgrounds to generate important knowledge to support the design of culturally sensitive services. Finally, the question of whether adapted nonpreference-based behavior can be reversed by the implementation of a preference-based leisure program intended to preserve and promote the well-being of people receiving ADS should be investigated.

## Supplemental Material

Supplemental Material - “This is Slowly Becoming my Interest…”: The Understanding of Leisure and Preferences for Leisure Activities of People Receiving Adult Day Services Click here for additional data file. Supplemental Material for “This is Slowly Becoming my Interest…”: The Understanding of Leisure and Preferences for Leisure Activities of People Receiving Adult Day Services by Mike Rommerskirch-Manietta, Christina Manietta, Daniel Purwins, Kimberly Van Haitsma, Katherine M. Abbott and Martina Roes in Research on Aging
