# Supplementary material for: “This is Slowly Becoming my Interest…”: The Understanding of Leisure and Preferences for Leisure Activities of People Receiving Adult Day Services
Source: Res Aging. 2023 Dec 12;46(3-4):210–27. doi: 10.1177/01640275231221162 (PMC10868149; doi:10.1177/01640275231221162)

Supplementary Material Table 1: Consolidated criteria for reporting qualitative research (COREQ): A 32-item checklist for interviews and focus groups

Item No.	Guiding questions/description-	Reported on page #
**Domain 1: Research team and reflexivity**		
Personal Characteristics		
1. Interviewer/facilitator	Which author/s conducted the interviews or focus groups?	7
2. Credentials	What were the researcher's credentials (e.g., PhD, MD)?	1; 7
3. Occupation	What was the researcher's occupation at the time of the study?	1; 7
4. Gender	Was the researcher male or female?	7
5. Experience and training	What experience or training did the researcher have?	7
Relationship with participants		
6. Relationship established	Was a relationship established prior to study commencement?	NA
7. Participant knowledge of the interviewer	What did the participants know about the researcher (e.g., personal goals, reasons for conducting the research)?	NA
**Domain 2: Study design**		
Theoretical framework		
9. Methodological orientation and theory	What methodological orientation was claimed to underlie the study (e.g., grounded theory, discourse analysis, ethnography, phenomenology, content analysis)?	6-7
Participant selection		
10. Sampling	How were participants selected (e.g., purposive, convenience, consecutive, snowball)?	6-7
11. Method of approach	How were participants approached (e.g., face-to-face, by telephone, by mail, by email)?	6-7
12. Sample size	How many participants were included in the study?	9
13. Nonparticipation	How many people refused to participate or dropped out? What were their reasons for doing so?	NA
Setting		
14. Setting of data collection	Where was the data collected (e.g., home, clinic, workplace)?	7
15. Presence of nonparticipants	Was anyone else present other than the participants and researchers?	7
16. Description of sample	What are the important characteristics of the sample (e.g., demographic data, dates)?	Table 2
Data collection		
17. Interview guide	Were questions, prompts, or guides provided by the authors? Were the guidelines pilot tested?	6-9; Supp. Table 2
18. Repeat interviews	Were repeat interviews conducted? If yes, how many?	NA
19. Audio/visual recording	Did the research use audio or visual recording to collect the data?	6-9
20. Field notes	Were field notes made during and/or after the interviews or focus groups?	7
21. Duration	What was the duration of the interviews or focus groups?	9
22. Data saturation	Was data saturation discussed?	9
23. Transcripts returned	Were transcripts returned to participants for comment and/or correction?	NA
**Domain 3: Analysis and findings**		
Data analysis		
24. Number of data coders	How many data coders coded the data?	8-9

Item No.	Guiding questions/description-	Reported on page #
25. Description of the coding tree	Did authors provide a description of the coding tree?	Supp. Figure 1
26. Derivation of themes	Were themes identified in advance or derived from the data?	8-9
27. Software	What software, if applicable, was used to manage the data?	8-9
28. Participant checking	Did participants provide feedback on the findings?	NA
Reporting		
29. Quotations presented	Were participant quotations presented to illustrate the themes/findings? Was the source of each quotation identified (e.g., by participant number)?	9-18
30. Consistency of data and findings	Was there consistency between the data presented and the findings?	9-18
31. Clarity of major themes	Were major themes presented clearly in the findings?	9-18
32. Clarity of minor themes	Was there a description of diverse cases or discussion of minor themes?	9-18

Supplementary Material Table 2: Interview guideline

Part 1: Leisure in general	
**Initial question**	**Follow-up questions**
- **What do you have in mind when you think about leisure?**	- *When do you engage in leisure?*<br>- *When do you not engage in leisure?*

Part 2: Leisure activities in general	
**1. Guiding question**	**Follow-up questions**
- **In what leisure activities do you engage?**<br>*Note to check:*<br>☐ ......<br>☐ ......<br>☐	- *What do you associate with these activities?*<br>- *Are there any leisure activities in which you would like to engage more often?*
**2. Guiding question**	**Follow-up questions**
- **Have your leisure activities changed over the course of your life?**	- *How have your leisure activities changed?*<br>- *What was the trigger for the change?*<br>*Follow-up questions and check if applicable:*<br>☐ *Aging*<br>☐ *Retirement*<br>☐ *Care needs/acute diseases*<br>☐ *Environment*<br>☐ *Social aspects (loss of partners, friends)*

3. Guiding question	Follow-up questions
- **What leisure activities would you like to try?**	*Follow-up questions and check if applicable:*<br>☐ *Active/passive*<br>☐ *Outdoor/indoor*<br>☐ *Groups/single*<br>☐ *Challenge/mastery*

4. Guiding question	Maintenance and control questions
- **How does receiving ADS affect your leisure activities?**	- *I haven't quite figured this out yet, could you explain it to me a little more....*<br>- *What do you mean by...*<br>- *How can I imagine that?*<br>- *Can you give an example to illustrate this point?*

Part 3: Leisure activities in the ADS

5. Guiding question	Maintenance and control questions
- **To what extent does receiving ADS count as a form of leisure for you?**	- *I haven't quite figured this out yet, could you explain it to me a little more....*<br>- *What do you mean by ...*<br>- *How can I imagine that?*<br>- *Can you give an example to illustrate this point?*<br>**If receiving ADS is not leisure, then continue by asking the following questions:**<br>- *Why does the visit not count as leisure for you?*<br>- - Are there individual activities within the ADS that do count as leisure for you?<br>**Proceed to question 7 without using the word leisure and add the following questions:**<br>- Are these activities not leisure activities for you?<br>- What are these activities for you?<br>**Then, continue asking the questions without using the word leisure**

6. Guiding question	
- **What activities in the ADS do you consider to be leisure activities?**	

7. Guiding question	Follow-up questions
- **In what leisure activities in the ADS do you participate?**<br>- **In what leisure activities in the ADS do you not participate?**	- *Why do you participate in these leisure activities?*<br>In case of discrepancies with the answers to guiding question 1, ask the following:<br>- How do the leisure activities in which you engage at home differ from those in the ADS?<br>- *Why don't you participate in these leisure activities?*

8. Guiding question	Follow-up question
- **What leisure activities would you like to try here at the ADS?**	*Follow-up questions and check if applicable:*<br>☐ *Active/passive*<br>☐ *Outdoor/indoor*<br>☐ *Croups/single*<br>☐ *Challenge/mastery*

9. Guiding question	Follow-up questions
- **What leisure activities would you like to see in the ADS?**	- *Why do you want these leisure activities?*<br>- *What would it mean to you if your preferred leisure activities were offered in the ADS?*<br>- *What conditions would have to be in place here for you to be able to engage in these leisure activities?*

Closing questions
- We have now talked a lot about your leisure and leisure activities. If you were now to summarize this topic again in one word, what is leisure for you?<br>- Is there anything else you would like to say/add that we haven't addressed?

Supplementary Material Figure 1: Illustration of coding

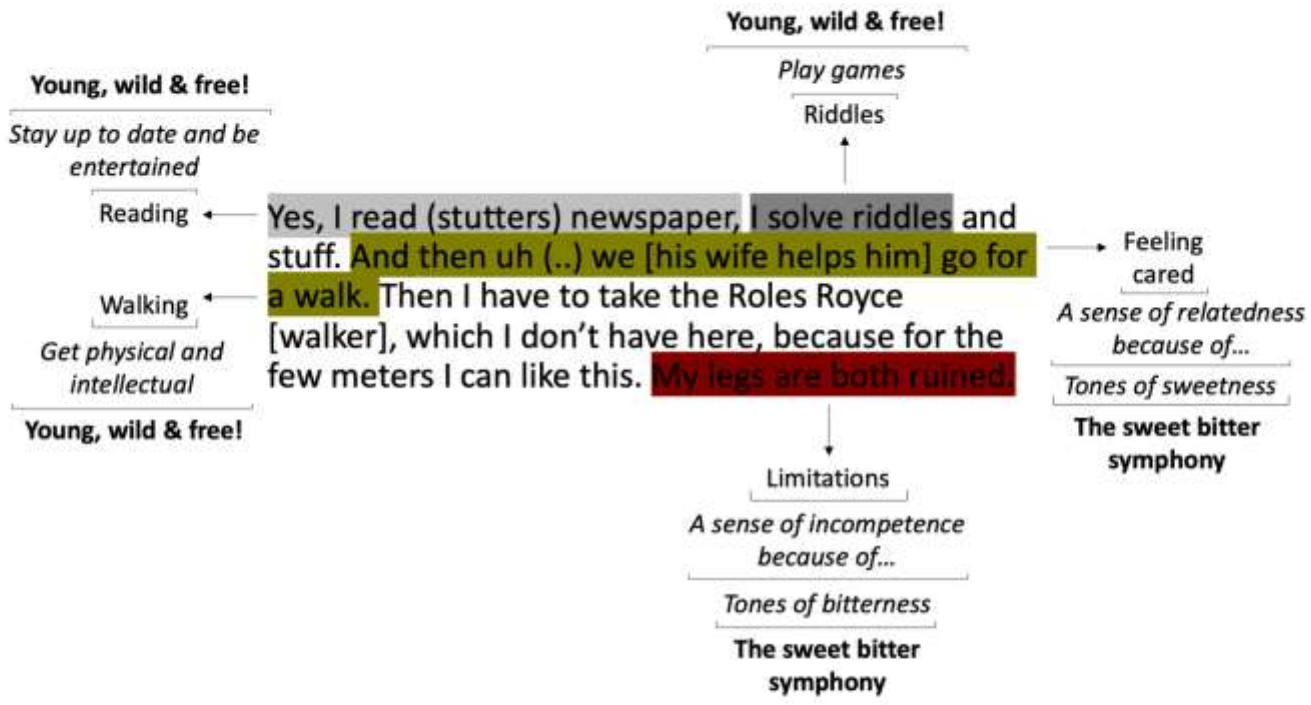

Supplement: Supplemental Material - “This is Slowly Becoming my Interest…”: The Understanding of Leisure and Preferences for Leisure Activities of People Receiving Adult Day Services [file sj-pdf-1-roa-10.1177_01640275231221162.pdf]
